# Mesosystem of mental health services and support, its actors and their collaborations—a study protocol for an integrative review

**DOI:** 10.1136/bmjopen-2024-089424

**Published:** 2025-09-23

**Authors:** Tove Törnqvist, Patrik Rytterström, Aricca Van Citters, Ingrid Rystedt

**Affiliations:** 1 Department of Health, Medicine and Caring Sciences (HMV), Linköping University, Linkoping, Sweden; 2 The Dartmouth Institute for Health Policy and Clinical Practice, Geisel School of Medicine at Dartmouth, Lebanon, New Hampshire, USA

**Keywords:** MENTAL HEALTH, PUBLIC HEALTH, Review, Health Services

## Abstract

**Introduction:**

Psychiatric conditions require long-term maintenance, and people with mental illness are often involved in care provided by multiple service providers. At the same time, reports indicate that people with mental illness and comorbid physical health conditions often fail to get their needs met in current systems. For example, coordinated support within the mesosystem is rarely available, and mesosystem fragmentation often results in individual suffering as well as inefficient use of societal resources. Meanwhile, integrated services, where collaboration is important, have shown superior effectiveness compared with services and support delivered in parallel silos. This contradictory image needs further investigation, and there is a need for a better understanding of the dynamics and complexities of collaboration within the mesosystem.

**Methods and analysis:**

This review aims to map the mesosystem for services and support for people with mental illnesses. The review will seek to answer questions such as who are the actors (formal and informal) in the mental health services and support mesosystem, and how are collaborations among actors organised and implemented? To synthesise qualitative, quantitative and mixed methods findings, it uses a systematic integrative methodology. Searches will be conducted in PubMed, PsycINFO, CINAHL and Web of Science between 17th June and 21st 2024. Data will be extracted and synthesised using Covidence software. Two blinded researchers will make determinations regarding the inclusion and exclusion of articles. The subsequent process of analysis will be jointly conducted by the whole research team. Preferred Reporting Items for Systematic Reviews and Meta-Analyses tools for quality assessments will be used.

**Ethics and dissemination:**

Articles included in the review will be analysed through an ethical lens, making sure that ethical considerations have been made where applicable. The study will result in a scientific publication in a peer-reviewed journal.

**Trial registration number:**

The study is registered in PROSPERO, no CRD42024543891.

**STRENGTHS AND LIMITATIONS OF THIS STUDY**

- The integrative review approach allows inclusion of both empirical and theoretical literature, broadening the analytical scope.
- The method supports synthesis across disciplines, which is essential for mapping diverse actors in the mesosystem.
- Flexibility in study design inclusion enhances responsiveness to emerging and underrepresented perspectives.
- The inclusion of diverse study designs may complicate synthesis and limit comparability across findings.
- Language restrictions and database selection may have led to the omission of culturally specific models of collaboration and support.

## Introduction

 Health and social care services for people with mental illness frequently revolve around long-term management and maintenance of mental health symptoms and functional impairments. The long-term nature of many mental health conditions requires that treatment decisions and delivery of supportive services frequently involve both informal and formal providers and agencies. Moreover, people with mental illness often have significant knowledge and lived experience related to their illness and play an important role in shaping service delivery. The complex combination of long-term conditions and multiple providers requires collaboration between the actors providing mental health services and support to people with mental illness. However, the system of health and social care service is not always conducive to collaboration. As a result, people with mental illness risk not having all their needs met.

This review has its starting point in a Swedish context. For decades, national evaluations of care and support services in Sweden have found that people with mental illness and comorbid disorders, such as substance use, often end up between the current health and social care systems.^1^ At the same time, there is solid scientific evidence for the superior effectiveness of integrated treatments.^2^ Only rarely, however, is coordinated support readily available in communities. Fragmentation leads to individual suffering and inefficient use of societal resources. Collaboration is increasingly important, and the situation calls for research that aims to broaden the perspective and learn from the international scope of literature describing integrated health and social support services.

The policy strategy ‘Health in all policies’ (HiAP)^3^ emphasises that population health and health equity are dependent on synergies across all policy areas and across all welfare sectors. By means of a systematic review, in part inspired by HiAP, this project aims to map the mesosystem of mental health services and support: mesosystem actors, collaboration and integrated initiatives among actors. In addition, the review will explore modes of collaboration and evaluate the respective roles of coproduction, interprofessional work, recovery principles and community involvement and their importance in the mesosystem of services for mental health. Nelson, Batalden and Godfrey characterise the mesosystem as: *‘an interrelated set of peer microsystems that provide care to certain populations or support the care provided to these populations*’ ^4^ [p. 203]. As such, the mesosystem requires an active dialogue between actors in the mesosystem, such as outpatient mental health clinics and rehabilitation centres. An important role of the mesosystem is indeed to transfer information between microsystems and between microsystems and the macrosystem.^5^

A necessary starting point for successful reorganisation and improvements is to characterise and thoroughly understand collaboration in the existing mesosystem in which mental healthcare and support already occur.^6^ As an example of a context where such knowledge is needed, in Sweden, there is an ongoing process of redesigning health services toward an increasing degree of integrated care (in Swedish nära vård, ‘close care’).^7^ Collaboration, and the four perspectives—coproduction, interprofessional work, recovery principles and community involvement—have the potential to guide this Swedish process towards integrated care, and to equip overall health and social care systems to sustainably provide mental health services and support functions for people with mental illness. The term ‘nära vård’ does not in itself recognise the importance of social services (beyond health services) and the society at large. Such actors may play intricate roles in supporting coproduction, interprofessional work, recovery principles and community involvement.

## Methods and analysis

This integrative review aims to map the full range of actors (or partners) that collectively make up the mesosystem of mental health services and support, how these actors collaborate, and how the collaborative services provided by the mesosystem impact mental health outcomes. A subsidiary aim is to explore whether and how the mesosystem of mental health services and support may be characterised or described by aspects of coproduction, interprofessional work, recovery principles and community involvement. The mesosystem is defined as *‘an interrelated set of peer microsystems that provide care to certain populations or support the care provided to these populations*’ (2, p. 203).

Specific research questions:

- Who are the actors (formal and informal) that collaborate in the mental health services and support mesosystem?
- How are collaborations among actors organised and implemented?
- Whether and how mesosystem activities incorporate elements of (1) Coproduction, (2) Interprofessional work, (3) Recovery principles and (4) Community involvement, and how can this inform future mesosystem design?

We anticipate that the research questions will generate a diverse set of data, including a variation of methodologies, contexts, use of theoretical frameworks and so on. This review will use an integrative design^8 9^ to synthesise findings from this diverse set of qualitative, quantitative and mixed methods studies.^8 9^ An integrative review aims to synthesise empirical and theoretical literature and is thus suitable when the set of articles covers diverse methodologies.^8^ The researchers will objectively critique, summarise and draw conclusions.

The systematic integrative review includes five stages:^8^ (1) Identification of the question or problem (see above aim), (2) Structured systematic search of international literature (with optimised search strategies and inclusion and exclusion criteria, see below), (3) Evaluation of the quality of included publications and (4) Data analysis, including thorough interpretation of primary sources, along with synthesis of extracted data. This process relies on the creation of tables of data to enable subsequent systematic analysis and inference. The final step (5) Involves presenting and explaining results of the review in workshops, scientific articles, reports and policy briefs.

### Data collection

We intend to search the following electronic bibliographic databases: PubMed, PsycInfo, CINAHL and Web of Science (science and social science citation index) as these are covering subjects relevant to this review. More specifically, PubMed provides access to high-quality research in medicine, psychiatry and biomedicine through the MEDLINE database. PsycInfo is the leading database for psychology and mental health and contains many articles not indexed in PubMed or Web of Science. CINAHL focuses on nursing, occupational therapy, physiotherapy and other healthcare professions and includes research on non-medical interventions like mindfulness, counselling and lifestyle changes. Web of Science covers a wide range of disciplines, useful for capturing cross-sectoral perspectives on mental health.

The search strategy, see table 1, will be developed in collaboration with librarians and include terms related to mental health, providers of care and collaboration. Search terms will be adapted for use in the respective bibliographic databases, combined with database-specific filters where these are available. Language is to be restricted to English, Danish, Norwegian and Swedish, as these languages are understood by three or more members of the research team. Studies published between January 2014 and June 2024 will be retrieved. The 10-year window focuses on the most recent and relevant studies, while allowing for the identification of a manageable volume of studies. The same searches will be re-run just prior to the final analyses, and potential further relevant studies will then be included.

**Table 1:** The search strategy is structured according to the PICO model (Population, Intervention, Comparison and Outcome) with three blocks^10^

Population	People with mental illness
Intervention	Mental health services, support and collaboration among actors. Mental health services and support and elements in mesosystem activities, such as: (1) Coproduction, (2) Interprofessional work, (3) Recovery principles and (4) Community involvement
Comparison	Not used
Outcome	Beyond describing the specific aspect of collaboration, the search will not focus on specific outcomes

The search strategy is developed in PubMed and contains relevant free text terms that have been converted to medical subject heading terms. Once the search strategy was developed for PubMed, it was modified for the other databases. See online supplemental appendix 1 for full version of the search string used in PubMed.

We will include empirical, qualitative and quantitative research studies. We will exclude studies such as reviews (in any form), study protocols and other non-research articles, such as opinion pieces, commentaries and conference papers. Hypothetical pieces representing expressions of what respondents ‘wish will be’ (speculatively or hypothetically) in the future will be excluded. Reports from regional, national or authorities, such as WHO, will be included if they are clearly empirical; otherwise, they will be excluded. See tables2  3 for detailed inclusion and exclusion criteria.

**Table 2:** Inclusion criteria

Inclusion criteria	Description
Mesosystem	Focus on at least two actors, or, in another way, make it clear that results pertain to the mesosystem level (and not the microsystem level)
Entities	Focus on functions, actions and/or services provided by two or more actors to promote mental health and/or treat mental illness
Mental health	Focus on the mental health of individuals, that is, not only, for example, the physical health of persons with mental illness (populations with comorbidities, not exclusion criteria, as long as their mental health is the focus)
Language	English, Swedish, Norwegian and Danish (ie, languages that at least three members of the research team can read and understand). Not enough with only an abstract in English

**Table 3:** Exclusion criteria

Exclusion criteria	Description
Reviews	Review articles (of any type), including meta-syntheses
Non-research articles	Such as opinion pieces, commentaries and conference papers. Hypothetical pieces representing expressions of what respondents ‘wish will be’ (speculatively or hypothetically) in the future are excluded
Protocols	Study protocols, where the study has not yet been implemented
Age of participants/target group	Focusing on individuals younger than 18. However, label age overlaps to later decide what to do with age overlap articles (both younger and those from 18 years)
Comorbidities	When focusing on the comorbidity itself and not the mental illness (however, if participants with comorbidity but the study aim is related to their mental health, this is not an exclusion criterion)
COVID-19	Focusing exclusively on temporary support put in place because of the situation during the COVID-19 pandemic (if research was conducted during the pandemic but had a clear long-term ‘aim’ beyond COVID-19, this is not an exclusion criterion)
No collaboration	The article describes fewer than two entities and/or does not portray any form of collaboration within the mesosystem (in the aims, methods or results)
Perceptions (of mental illness)	Primarily focusing on how staff, community members or others perceive mental illness, or persons with mental illness, or challenges related to having mental illness
Experiences (of mental illness)	Primarily focusing on patient experiences of mental illness, for example, stigma
Treatment studies	Only focusing on comparing x number of tx’s within the same actor/provider (also collaborations within the same actors/provider, ie, same microsystem), strict focus on treatment
Foreign language	Articles in a language other than English, Swedish, Norwegian and Danish
Wrong population: diagnosis	Does not focus on mental illness (specifically dementia)

### Data extraction and synthesis

The computer-assisted searches drawing on the comprehensive sets and combinations of search terms will be implemented in PubMed, Web of Science, CINAHL and PsychInfo. The search processes are documented, and inclusion and exclusion criteria are listed in tables2  3. Reference lists from systematic reviews may still be explored to identify relevant empirical research articles. EndNote software will be used to identify duplicates in searches from different databases. Covidence software will be used for screening and data extraction.

Two researchers will assess each identified primary source. They will be blinded to the determinations made by the other researcher. When contrasting decisions regarding inclusion or exclusion are made, one or two additional researchers (similarly blinded) will examine the source for inclusion or exclusion. Unclear cases will be thoroughly discussed in the research group. Subsequently, the quality of included sources will be assessed with quality criteria instruments, such as Preferred Reporting Items for Systematic Reviews and Meta-Analyses. This step will be made collaboratively in the research group. If and when primary sources are excluded in this phase, such decisions will be documented. Similarly, the group will discuss the risk of bias due to, for example, missing data. These reflections will subsequently be described as important context and potential limitations for the study.

Data collection and data reduction will begin with the identification and classification of suitable subgroupings (with clear relevance to the aim) to facilitate and organise subsequent analyses.^8 9^ Preliminary subgroups will be created by geographic region. Other subgrouping may also be relevant, depending on the findings. All data will be retrieved and organised by subgroup. Initially, this data will be compiled into one spreadsheet for each primary source, including all retrieved data, sorted by subgroup.

In order to integrate findings based on primary sources with a multitude of methodologies, this review will include processes for data reduction, data display, data comparison, conclusion drawing and verification, and presentation for each step in the data analysis, as suggested by Whittemore and Knafl.^8^

### Ethics and dissemination

Ethical approvals are not applicable to the review. However, articles included in the review will be analysed to ensure that ethical considerations have been disclosed, where applicable.

The study will result in at least one manuscript to be submitted to a peer-reviewed journal within the field of mental health and mental illness. In addition, the review will be disseminated to the funders: the Swedish Research Council for Health, Working Life and Welfare (Forte).

## Supplementary material

10.1136/bmjopen-2024-089424 online supplemental file 1
