# Supplementary material for: Mesosystem of mental health services and support, its actors and their collaborations—a study protocol for an integrative review
Source: BMJ Open. 2025 Sep 23;15(9):e089424. doi: 10.1136/bmjopen-2024-089424 (PMC12458866; doi:10.1136/bmjopen-2024-089424)
Supplement: online supplemental file 1 [file bmjopen-15-9-s001.docx]

Appendix 1 – search in MEDLINE/PubMed

20240618

1552 hits

(“mental disorders”[majr:noexp] OR “mood disorders”[mesh] OR “schizophrenia spectrum and other psychotic disorders”[mesh] OR “mental illness*”[ti] OR “mental disorder*”[ti] OR “mental disease*”[ti] OR bipolar[tiab] OR manic-depress*[tiab] OR “manic disorder*”[tiab] OR depression[tiab] OR depressive[tiab] OR dysthymi*[tiab] OR schizophreni*[tiab] OR schizoaffective[tiab] OR psychotic[tiab] OR psychoses[tiab] OR paranoi*[tiab])

AND

(”mental health services”[mesh] OR “mental health associations”[mesh] OR “social welfare”[mesh] OR "social support"[mesh:noexp] OR "psychosocial support systems"[mesh] OR “social work, psychiatric” [mesh] OR "residential facilities"[mesh:noexp] OR "group homes"[mesh] OR "halfway houses"[mesh] OR "assisted living facilities"[mesh] OR "community mental health services"[mesh] OR "employment, supported"[mesh] OR ”mental health service”[tiab:~3] OR ”mental health services”[tiab:~3] OR “mental health association*”[tiab] OR “mental health support”[tiab] OR “mental health care”[tiab] OR “welfare service”[tiab] OR “practice models”[tiab] OR “treatment models”[tiab] OR “social service*”[tiab] OR “housing service*”[tiab] OR “social welfare”[tiab] OR "psychosocial support system*"[tiab] OR “social support system*”[tiab] OR “psychiatric social work”[tiab] OR “social psychiatric work”[tiab] OR “psychiatric social service*”[tiab] OR “social psychiatric service*”[tiab] OR "residential facility"[tiab:~3] OR "residential facilities"[tiab:~3] OR "group home*"[tiab] OR "halfway house*"[tiab] OR "assisted living facilit*"[tiab] OR "assertive community treatment"[tiab] OR "supported employment"[tiab] OR "supported housing”[tiab])

AND

((“intersectoral collaboration”[mesh] OR “cooperative behavior”[mesh] OR “delivery of health care, integrated”[mesh] OR “collaboration care”[tiab:~3] OR “collaboration mental health”[tiab:~3] OR “collaboration psychiatric”[tiab:~3] OR “collaboration service”[tiab:~3] OR “collaboration services”[tiab:~3] OR ”collaboration strategy”[tiab:~3] OR ”collaboration strategies”[tiab:~3] OR ”collaboration practice”[tiab:~3] OR ”collaboration practices”[tiab:~3] OR “collaboration team”[tiab:~3] OR “collaboration teams”[tiab:~3] OR ”collaboration work”[tiab:~3] OR ”collaboration providers”[tiab:~3] OR ”collaboration community”[tiab:~3] OR ”collaboration communities”[tiab:~3] OR “collaboration professions”[tiab:~3] OR ”collaboration professional”[tiab:~3] OR ”collaboration professionals”[tiab:~3] OR ”collaboration stakeholders”[tiab:~3] OR ”collaboration agencies”[tiab:~3] OR ”collaboration organizations”[tiab:~3] OR ”collaboration organizational”[tiab:~3] OR “collaboration organisations”[tiab:~3] OR ”collaboration organisational”[tiab:~3] OR ”collaboration intersectoral”[tiab:~3] OR “cooperation care”[tiab:~3] OR “cooperation mental health”[tiab:~3] OR “cooperation psychiatric”[tiab:~3] OR “cooperation service”[tiab:~3] OR “cooperation services”[tiab:~3] OR ”cooperation strategy”[tiab:~3] OR ”cooperation strategies”[tiab:~3] OR ”cooperation practice”[tiab:~3] OR ”cooperation practices”[tiab:~3] OR “cooperation team”[tiab:~3] OR “cooperation teams”[tiab:~3] OR ”cooperation work”[tiab:~3] OR ”cooperation providers”[tiab:~3] OR ”cooperation community”[tiab:~3] OR ”cooperation communities”[tiab:~3] OR “cooperation professions”[tiab:~3] OR ”cooperation professional”[tiab:~3] OR ”cooperation professionals”[tiab:~3] OR ”cooperation stakeholders”[tiab:~3] OR ”cooperation agencies”[tiab:~3] OR ”cooperation organizations”[tiab:~3] OR ”cooperation organizational”[tiab:~3] OR “cooperation organisations”[tiab:~3] OR ”cooperation organisational”[tiab:~3] OR ”cooperation intersectoral”[tiab:~3] OR “co-operation care”[tiab:~3] OR “co-operation mental health”[tiab:~3] OR “co-operation psychiatric”[tiab:~3] OR “co-operation service”[tiab:~3] OR “co-operation services”[tiab:~3] OR ”co-operation strategy”[tiab:~3] OR ”co-operation strategies”[tiab:~3] OR ”co-operation practice”[tiab:~3] OR ”co-operation practices”[tiab:~3] OR “co-operation team”[tiab:~3] OR “co-operation teams”[tiab:~3] OR ”co-operation work”[tiab:~3] OR ”co-operation providers”[tiab:~3] OR ”co-operation community”[tiab:~3] OR ”co-operation communities”[tiab:~3] OR “co-operation professions”[tiab:~3] OR ”co-operation professional”[tiab:~3] OR ”co-operation professionals”[tiab:~3] OR ”co-operation stakeholders”[tiab:~3] OR ”co-operation agencies”[tiab:~3] OR ”co-operation organizations”[tiab:~3] OR ”co-operation organizational”[tiab:~3] OR “co-operation organisations”[tiab:~3] OR ”co-operation organisational”[tiab:~3] OR ”co-operation intersectoral”[tiab:~3] OR “collaborations care”[tiab:~3] OR “collaborations mental health”[tiab:~3] OR “collaborations psychiatric”[tiab:~3] OR “collaborations service”[tiab:~3] OR “collaborations services”[tiab:~3] OR ”collaborations strategy”[tiab:~3] OR ”collaborations strategies”[tiab:~3] OR ”collaborations practice”[tiab:~3] OR ”collaborations practices”[tiab:~3] OR “collaborations team”[tiab:~3] OR “collaborations teams”[tiab:~3] OR ”collaborations work”[tiab:~3] OR ”collaborations providers”[tiab:~3] OR ”collaborations community”[tiab:~3] OR ”collaborations communities”[tiab:~3] OR “collaborations professions”[tiab:~3] OR ”collaborations professional”[tiab:~3] OR ”collaborations professionals”[tiab:~3] OR ”collaborations stakeholders”[tiab:~3] OR ”collaborations agencies”[tiab:~3] OR ”collaborations organizations”[tiab:~3] OR ”collaborations organizational”[tiab:~3] OR “collaborations organisations”[tiab:~3] OR ”collaborations organisational”[tiab:~3] OR ”collaborations intersectoral”[tiab:~3] OR “cooperations care”[tiab:~3] OR “cooperations mental health”[tiab:~3] OR “cooperations psychiatric”[tiab:~3] OR “cooperations service”[tiab:~3] OR “cooperations services”[tiab:~3] OR ”cooperations strategy”[tiab:~3] OR ”cooperations strategies”[tiab:~3] OR ”cooperations practice”[tiab:~3] OR ”cooperations practices”[tiab:~3] OR “cooperations team”[tiab:~3] OR “cooperations teams”[tiab:~3] OR ”cooperations work”[tiab:~3] OR ”cooperations providers”[tiab:~3] OR ”cooperations community”[tiab:~3] OR ”cooperations communities”[tiab:~3] OR “cooperations professions”[tiab:~3] OR ”cooperations professional”[tiab:~3] OR ”cooperations professionals”[tiab:~3] OR ”cooperations stakeholders”[tiab:~3] OR ”cooperations agencies”[tiab:~3] OR ”cooperations organizations”[tiab:~3] OR ”cooperations organizational”[tiab:~3] OR “cooperations organisations”[tiab:~3] OR ”cooperations organisational”[tiab:~3] OR ”cooperations intersectoral”[tiab:~3] OR “co-operations care”[tiab:~3] OR “co-operations mental health”[tiab:~3] OR “co-operations psychiatric”[tiab:~3] OR “co-operations service”[tiab:~3] OR “co-operations services”[tiab:~3] OR ”co-operations strategy”[tiab:~3] OR ”co-operations strategies”[tiab:~3] OR ”co-operations practice”[tiab:~3] OR ”co-operations practices”[tiab:~3] OR “co-operations team”[tiab:~3] OR “co-operations teams”[tiab:~3] OR ”co-operations work”[tiab:~3] OR ”co-operations providers”[tiab:~3] OR ”co-operations community”[tiab:~3] OR ”co-operations communities”[tiab:~3] OR “co-operations professions”[tiab:~3] OR ”co-operations professional”[tiab:~3] OR ”co-operations professionals”[tiab:~3] OR ”co-operations stakeholders”[tiab:~3] OR ”co-operations agencies”[tiab:~3] OR ”co-operations organizations”[tiab:~3] OR ”co-operations organizational”[tiab:~3] OR “co-operations organisations”[tiab:~3] OR ”co-operations organisational”[tiab:~3] OR ”co-operations intersectoral”[tiab:~3] OR ”collaborative care”[tiab:~3] OR “collaborative mental health”[tiab:~3] OR “collaborative psychiatric”[tiab:~3] OR “collaborative service”[tiab:~3] OR ”collaborative services”[tiab:~3] OR ”collaborative strategy”[tiab:~3] OR ”collaborative strategies”[tiab:~3] OR ”collaborative practice”[tiab:~3] OR ”collaborative practices”[tiab:~3] OR “collaborative team”[tiab:~3] OR “collaborative teams”[tiab:~3] OR ”collaborative work”[tiab:~3] OR ”cooperative care”[tiab:~3] OR “cooperative mental health”[tiab:~3] OR “cooperative psychiatric”[tiab:~3] OR “cooperative service”[tiab:~3] OR ”cooperative services”[tiab:~3] OR ”cooperative strategy”[tiab:~3] OR ”cooperative strategies”[tiab:~3] OR ”cooperative practice”[tiab:~3] OR ”cooperative practices”[tiab:~3] OR “cooperative team”[tiab:~3] OR “cooperative teams”[tiab:~3] OR ”cooperative work”[tiab:~3] OR ”co-operative care”[tiab:~3] OR “co-operative mental health”[tiab:~3] OR “co-operative psychiatric”[tiab:~3] OR “co-operative service”[tiab:~3] OR ”co-operative services”[tiab:~3] OR ”co-operative strategy”[tiab:~3] OR ”co-operative strategies”[tiab:~3] OR ”co-operative practice”[tiab:~3] OR ”co-operative practices”[tiab:~3] OR “co-operative team”[tiab:~3] OR “co-operative teams”[tiab:~3] OR ”co-operative work”[tiab:~3] OR team-work*[tiab] OR teamwork*[tiab] OR “integrated services”[tiab:~3] OR “integrated care”[tiab:~3] OR co-design[tiab])

NOT

((child[mesh] OR adolescent[mesh]) NOT adult[mesh])

NOT

(letter[pt] OR “case reports”[pt])

AND

("2013/01/01"[Date - Publication] : "3000"[Date - Publication])

AND

(English[language] OR Swedish[language] OR Norwegian[language] OR Danish[language])
