# Supplementary material for: Mesosystem of mental health services and support, its actors and their collaborations—a study protocol for an integrative review
Source: BMJ Open. 2025 Sep 23;15(9):e089424. doi: 10.1136/bmjopen-2024-089424 (PMC12458866; doi:10.1136/bmjopen-2024-089424)
Supplement: Supplementary file 2 [file bmjopen-2024-089424.reviewer_comments.pdf]

PEER REVIEW HISTORY

BMJ Open publishes all reviews undertaken for accepted manuscripts. Reviewers are asked to complete a checklist review form and are provided with free text boxes to elaborate on their assessment. These free text comments are reproduced below.

ARTICLE DETAILS

Title (Provisional)

The mesosystem of mental health services and support, its actors and their collaborations – A study protocol for an integrative review

Authors

Törnqvist, Tove; Rytterström, Patrik; Van Citters, Aricca; Rystedt, Ingrid

---

VERSION 1 - REVIEW

**Reviewer**	**1**
**Name**	**van Rensburg, André**
**Affiliation**	**University of KwaZulu-Natal, Centre for Rural Health**
**Date**	**03-Sep-2024**
**COI**	**None declared.**

This is a timely review, targeting a

**Reviewer**	**2**
**Name**	**Morgan, A**
**Affiliation**	**Glasgow Caledonian University**
**Date**	**22-Sep-2024**
**COI**	**None**

BMJ Open

The mesosystem of mental health services and support, its actors and their collaborations – A study protocol for an integrative review

Firstly, it is commended that this protocol describes an integrative review that focuses on determinants of health at the meso level of influence. So often, social scientists explore the most inner level of (determinants) often referred to as the micro. Throughout the document there is a lack of attention to defining concepts e.g meso, structured takjk definition of term

.g meso. The abstract could be improved so that it makes explicit why the research is important and why an integrative review is the best approach

The manuscript would benefit from a proof reading by a native English speaker as there is unusual phraseology throughout the protocol . For example, ‘Swedish persons’ probably better as ‘Swedish people the Swedish population, a small mistake that if corrected could improve the quality of the paper/. The English in general is ok

Main Text

‘‘the researchers will take an inclusive approach to publications while concurrently maintaining the highest standards of rigor.’

What does this actually mean. Please define an integrative review. Are you following a particular standard of protocol from Cochrane, EPPI centre?. What do you mean by inclusive approach is this to do with inclusion criteria or something else

PubMed, PsycInfo, CINAHL, and Web of Science (science and social science citation index)

What is the rationale for using these databases and platforms(PubMed is a platform as far as I know)why is Medline not included

1evaluation of the quality of included publications'

publications or studies which appraisal tool will you use

Studies published between January 2014 and May 2024. This is standard but you should state why you have opted for 10 years. Overall not a bad protocol but sometimes detail on methods is scant making it difficult to be explicit about what you intend to do why. and how

---

VERSION 1 - AUTHOR RESPONSE

Reviewer comments	Authors’ response
**Throughout the document there is a lack of attention to defining concepts e.g meso, structured takjk definition of term .g meso.**	**The concept of mesosystem is defined in the introduction. We have added the definition under ‘method and analysis’ as well.**
**The abstract could be improved so that it makes explicit why the research is important and why an integrative review is the best approach**	**The abstract has been updated with the intention of clarifying the need for an integrative review. For example, the introduction is shorter as an effort to be more straight forward.**
**The manuscript would benefit from a proof reading by a native English speaker as there is unusual phraseology throughout the protocol. For example, ‘Swedish persons’ probably better as ‘Swedish people the**	**One of the native English-speaking authors have proofread the manuscript once more. Hopefully, we have reduced the unusual phraseologies.**

Swedish population, a small mistake that if corrected could improve the quality of the paper/. The English in general is ok	
‘‘the researchers will take an inclusive approach to publications while concurrently maintaining the highest standards of rigor.’ What does this actually mean. Please define an integrative review. Are you following a particular standard of protocol from Cochrane, EPPI centre?. What do you mean by inclusive approach is this to do with inclusion criteria or something else	Thank you for pointing this out. We have added information on the integrative review in hope of clarifying our choice and why we think it is the best choice of method. In terms of inclusive approach, we understand the vague phraseology and have chosen to remove ii as our approach, to include multiple methods, are described elsewhere.
PubMed, PsycInfo, CINAHL, and Web of Science (science and social science citation index).<br>What is the rationale for using these databases and platforms (PubMed is a platform as far as I know) why is Medline not included	We have clarified that PubMed is a platform based on MEDLINE.
Evaluation of the quality of included publications'<br>publications or studies which appraisal tool will you use<br>Studies published between January 2014 and May 2024. This is standard but you should state why you have opted for 10 years.	A valid point, we have added information on how we assessed quality. In addition, we have stated why we opted for 10 years.
Overall not a bad protocol but sometimes detail on methods is scant making it difficult to be explicit about what you intend to do why. and how	Thank you, we are pleased to hear. We also see your point and have added more details. E.g. we have added a new appendix detailing our inclusion and exclusion criteria. Hopefully these details will improve the protocol.
